# Supplementary material for: Sorting at embryonic boundaries requires high heterotypic interfacial tension
Source: Nat Commun. 2017 Jul 31;8:157. doi: 10.1038/s41467-017-00146-x (PMC5537356; doi:10.1038/s41467-017-00146-x)
Supplement: Supplementary file 2 — Supplementary Software 1 [file 41467_2017_146_MOESM2_ESM.zip › PottsModel/SrcPottsModel/doc/model/Constants.html]

Constants


JavaScript is disabled on your browser.


Skip navigation links


- Overview
- Package
- Class
- Use
- Tree
- Deprecated
- Index
- Help

- Prev Class
- Next Class

- Frames
- No Frames

- All Classes

- Summary:
- Nested |
- Field |
- Constr |
- Method

- Detail:
- Field |
- Constr |
- Method


model

## Class Constants

- java.lang.Object
- - model.Constants

- ---

    

  ```
  public class Constants
  extends java.lang.Object
  ```

  Reads Constants from config file

  Author:
  :   Eleyine

- - ### Nested Class Summary

    Nested Classes

    | Modifier and Type | Class and Description |
    | --- | --- |
    | `static class` | `Constants.Model` |
    | `class` | `Constants.ParameterManager` Manages energy information related to this simulation. |
  - ### Field Summary

    Fields

    | Modifier and Type | Field and Description |
    | --- | --- |
    | `Constants.ParameterManager` | `params` |
  - ### Constructor Summary

    Constructors

    Constructor and Description

    `Constants()`

    Load Constants with default params (see source code for default values).

    `Constants(java.lang.String pConfigFilePath)`

    Load Constants from custom configuration file (missing fields populated
    with default values).

    `Constants(java.lang.String pConfigFilePath,
    java.lang.String pSimulationName)`

    Load Constants from custom configuration file and give simulation a name.
  - ### Method Summary

    All Methods Instance Methods Concrete Methods

    | Modifier and Type | Method and Description |
    | --- | --- |
    | `void` | `createPaths()` |
    | `void` | `disableSaving()` |
    | `java.lang.String` | `getBaseSimulationName()` |
    | `java.lang.String` | `getConfigFilename()` |
    | `int` | `getCsvWriteFrequency()` |
    | `java.lang.String` | `getCurrentPropertiesFilename()` |
    | `java.lang.String` | `getInitialLatticeFilename()` |
    | `java.lang.String` | `getLatticeDirectory()` |
    | `java.lang.String` | `getLatticeFilename(int numberMCS)` |
    | `int` | `getLatticeWriteFrequency()` |
    | `int` | `getMaxMcs()` |
    | `int` | `getNumberOfNeirestNeighbors()` |
    | `int` | `getPixelSize()` |
    | `java.util.Properties` | `getProperties()` |
    | `java.lang.String` | `getRootDirectory()` |
    | `int` | `getScreenHeight()` |
    | `int` | `getScreenWidth()` |
    | `java.lang.String` | `getSimulationName()` |
    | `java.lang.String` | `getSnapshotDirectory()` |
    | `java.lang.String` | `getSnapshotFilename(int numberMCS)` |
    | `int` | `getSnapshotWriteFrequency()` |
    | `java.lang.String` | `getStatsFilename()` |
    | `java.lang.String` | `getTimeStampString()` |
    | `boolean` | `isAppendToCSV()` |
    | `boolean` | `isAreaStatisticTracked()` |
    | `boolean` | `isAutomaticMode()` |
    | `boolean` | `isCellCoordinatesCSVStatisticTracked()` |
    | `boolean` | `isCellEnergyStatisticTracked()` |
    | `boolean` | `isCellShapeCSVStatisticTracked()` |
    | `boolean` | `isCsvSaved()` |
    | `boolean` | `isDebugMode()` |
    | `boolean` | `isDispersionIndexStatisticTracked()` |
    | `boolean` | `isEnabled()` |
    | `boolean` | `isGraphicalOutputEnabled()` |
    | `boolean` | `isHblStatisticTracked()` |
    | `boolean` | `isHMDStatisticTracked()` |
    | `boolean` | `isIsoperimetricQuotientStatisticTracked()` |
    | `boolean` | `isLatticeSaved()` |
    | `boolean` | `isNeighborsTracked()` |
    | `boolean` | `isNNStatisticTracked()` |
    | `boolean` | `isPerimeterStatisticTracked()` |
    | `boolean` | `isRandomCellTypes()` |
    | `boolean` | `isSaved()` Is anything being saved? |
    | `boolean` | `isScalePixelSize()` |
    | `boolean` | `isSnapshotsSaved()` |
    | `boolean` | `isSuccessfulSpinCopyAttemptsTracked()` |
    | `void` | `setAutomaticMode(boolean enabled)` |
    | `void` | `setCellCoordinatesCSVStatisticTracked(boolean pEnabled)` |
    | `void` | `setCellShapeCSVStatisticTracked(boolean pEnabled)` |
    | `void` | `setEnabled(boolean enabled)` |
    | `void` | `setGraphicalOutput(boolean enabled)` |
    | `void` | `setInitialLatticeFilename(java.lang.String path)` |
    | `void` | `setMaxMcs(int mcs)` |
    | `void` | `setPixelSize(int pixelSize)` |
    | `void` | `setPixelType(PixelShape.Type pType)` |
    | `void` | `setRandomCellTypes(boolean enabled)` |
    | `void` | `setRootDirectory(java.lang.String directory)` |
    | `void` | `setScalePixelSize(boolean scalePixelSize)` |
    | `void` | `setScreenHeight(int screenHeight)` |
    | `void` | `setScreenWidth(int screenWidth)` |
    | `void` | `setSimulationName(java.lang.String name)` |
    | `void` | `setSnapshotSaved(boolean enabled)` |
    | `java.lang.String` | `toString()` |

    - ### Methods inherited from class java.lang.Object

      `equals, getClass, hashCode, notify, notifyAll, wait, wait, wait`

- - ### Field Detail


    - #### params

      ```
      public Constants.ParameterManager params
      ```
  - ### Constructor Detail


    - #### Constants

      ```
      public Constants()
      ```

      Load Constants with default params (see source code for default values).


    - #### Constants

      ```
      public Constants(java.lang.String pConfigFilePath)
      ```

      Load Constants from custom configuration file (missing fields populated
      with default values).

      Parameters:
      :   `pConfigFilePath` - the configuration file path


    - #### Constants

      ```
      public Constants(java.lang.String pConfigFilePath,
                       java.lang.String pSimulationName)
      ```

      Load Constants from custom configuration file and give simulation a name.

      Parameters:
      :   `pConfigFilePath` -
      :   `name` -
  - ### Method Detail


    - #### getProperties

      ```
      public java.util.Properties getProperties()
      ```


    - #### toString

      ```
      public java.lang.String toString()
      ```

      Overrides:
      :   `toString` in class `java.lang.Object`


    - #### isSaved

      ```
      public boolean isSaved()
      ```

      Is anything being saved?

      Returns:
      :   whether at least one of the following is saved: simulation
          snapshots, statistics CSV, lattice in text format


    - #### isAutomaticMode

      ```
      public boolean isAutomaticMode()
      ```


    - #### setAutomaticMode

      ```
      public void setAutomaticMode(boolean enabled)
      ```


    - #### setGraphicalOutput

      ```
      public void setGraphicalOutput(boolean enabled)
      ```


    - #### isGraphicalOutputEnabled

      ```
      public boolean isGraphicalOutputEnabled()
      ```


    - #### getSimulationName

      ```
      public java.lang.String getSimulationName()
      ```


    - #### setSimulationName

      ```
      public void setSimulationName(java.lang.String name)
      ```


    - #### getTimeStampString

      ```
      public java.lang.String getTimeStampString()
      ```


    - #### getBaseSimulationName

      ```
      public java.lang.String getBaseSimulationName()
      ```


    - #### getMaxMcs

      ```
      public int getMaxMcs()
      ```


    - #### setMaxMcs

      ```
      public void setMaxMcs(int mcs)
      ```


    - #### isDebugMode

      ```
      public boolean isDebugMode()
      ```


    - #### isSnapshotsSaved

      ```
      public boolean isSnapshotsSaved()
      ```


    - #### isCsvSaved

      ```
      public boolean isCsvSaved()
      ```


    - #### isLatticeSaved

      ```
      public boolean isLatticeSaved()
      ```


    - #### isAppendToCSV

      ```
      public boolean isAppendToCSV()
      ```


    - #### getSnapshotWriteFrequency

      ```
      public int getSnapshotWriteFrequency()
      ```


    - #### getCsvWriteFrequency

      ```
      public int getCsvWriteFrequency()
      ```


    - #### getLatticeWriteFrequency

      ```
      public int getLatticeWriteFrequency()
      ```


    - #### isSuccessfulSpinCopyAttemptsTracked

      ```
      public boolean isSuccessfulSpinCopyAttemptsTracked()
      ```


    - #### isHblStatisticTracked

      ```
      public boolean isHblStatisticTracked()
      ```


    - #### isCellEnergyStatisticTracked

      ```
      public boolean isCellEnergyStatisticTracked()
      ```


    - #### isDispersionIndexStatisticTracked

      ```
      public boolean isDispersionIndexStatisticTracked()
      ```


    - #### isAreaStatisticTracked

      ```
      public boolean isAreaStatisticTracked()
      ```


    - #### isPerimeterStatisticTracked

      ```
      public boolean isPerimeterStatisticTracked()
      ```


    - #### isIsoperimetricQuotientStatisticTracked

      ```
      public boolean isIsoperimetricQuotientStatisticTracked()
      ```


    - #### isNNStatisticTracked

      ```
      public boolean isNNStatisticTracked()
      ```


    - #### isHMDStatisticTracked

      ```
      public boolean isHMDStatisticTracked()
      ```


    - #### getNumberOfNeirestNeighbors

      ```
      public int getNumberOfNeirestNeighbors()
      ```


    - #### isCellShapeCSVStatisticTracked

      ```
      public boolean isCellShapeCSVStatisticTracked()
      ```


    - #### setCellShapeCSVStatisticTracked

      ```
      public void setCellShapeCSVStatisticTracked(boolean pEnabled)
      ```


    - #### isCellCoordinatesCSVStatisticTracked

      ```
      public boolean isCellCoordinatesCSVStatisticTracked()
      ```


    - #### setCellCoordinatesCSVStatisticTracked

      ```
      public void setCellCoordinatesCSVStatisticTracked(boolean pEnabled)
      ```


    - #### isEnabled

      ```
      public boolean isEnabled()
      ```


    - #### setEnabled

      ```
      public void setEnabled(boolean enabled)
      ```


    - #### getPixelSize

      ```
      public int getPixelSize()
      ```


    - #### setPixelSize

      ```
      public void setPixelSize(int pixelSize)
      ```


    - #### isScalePixelSize

      ```
      public boolean isScalePixelSize()
      ```


    - #### setScalePixelSize

      ```
      public void setScalePixelSize(boolean scalePixelSize)
      ```


    - #### getScreenWidth

      ```
      public int getScreenWidth()
      ```


    - #### setScreenWidth

      ```
      public void setScreenWidth(int screenWidth)
      ```


    - #### getScreenHeight

      ```
      public int getScreenHeight()
      ```


    - #### setScreenHeight

      ```
      public void setScreenHeight(int screenHeight)
      ```


    - #### setRootDirectory

      ```
      public void setRootDirectory(java.lang.String directory)
      ```


    - #### getRootDirectory

      ```
      public java.lang.String getRootDirectory()
      ```


    - #### getSnapshotDirectory

      ```
      public java.lang.String getSnapshotDirectory()
      ```


    - #### getLatticeDirectory

      ```
      public java.lang.String getLatticeDirectory()
      ```


    - #### getStatsFilename

      ```
      public java.lang.String getStatsFilename()
      ```


    - #### getConfigFilename

      ```
      public java.lang.String getConfigFilename()
      ```


    - #### getCurrentPropertiesFilename

      ```
      public java.lang.String getCurrentPropertiesFilename()
      ```


    - #### getInitialLatticeFilename

      ```
      public java.lang.String getInitialLatticeFilename()
      ```


    - #### setInitialLatticeFilename

      ```
      public void setInitialLatticeFilename(java.lang.String path)
      ```


    - #### getLatticeFilename

      ```
      public java.lang.String getLatticeFilename(int numberMCS)
      ```


    - #### getSnapshotFilename

      ```
      public java.lang.String getSnapshotFilename(int numberMCS)
      ```


    - #### createPaths

      ```
      public void createPaths()
      ```


    - #### setSnapshotSaved

      ```
      public void setSnapshotSaved(boolean enabled)
      ```


    - #### disableSaving

      ```
      public void disableSaving()
      ```


    - #### setPixelType

      ```
      public void setPixelType(PixelShape.Type pType)
      ```


    - #### setRandomCellTypes

      ```
      public void setRandomCellTypes(boolean enabled)
      ```


    - #### isRandomCellTypes

      ```
      public boolean isRandomCellTypes()
      ```


    - #### isNeighborsTracked

      ```
      public boolean isNeighborsTracked()
      ```


Skip navigation links


- Overview
- Package
- Class
- Use
- Tree
- Deprecated
- Index
- Help

- Prev Class
- Next Class

- Frames
- No Frames

- All Classes

- Summary:
- Nested |
- Field |
- Constr |
- Method

- Detail:
- Field |
- Constr |
- Method
